# Effects of Specific Carob (*Ceratonia siliqua* L.) Liquid Concentrate on Glucose Metabolism in Subjects with Prediabetes: A Randomized Double-Blind Controlled Clinical Trial

**DOI:** 10.3390/nu18101521

**Published:** 2026-05-10

**Authors:** Silvia Pérez-Piñero, Juan Carlos Muñoz-Carrillo, Cristina Herrera-Fernández, Macarena Muñoz-Cámara, Almudena Hernández-Aliaga, Jon Echepare-Taberna, Vicente Ávila-Gandía, Francisco Javier López-Román

**Affiliations:** 1 Health Sciences Department, Universidad Católica San Antonio de Murcia (UCAM), Campus de los Jerónimos, Carretera de Guadalupe s/n, Guadalupe, E-30107 Murcia, Spain; sperez2@ucam.edu (S.P.-P.); cherrera@ucam.edu (C.H.-F.); mmunoz5@ucam.edu (M.M.-C.); ahernandez6@ucam.edu (A.H.-A.); echepare@ucam.edu (J.E.-T.); vavila@ucam.edu (V.Á.-G.); jlroman@ucam.edu (F.J.L.-R.); 2 Primary Care Research Group, Biomedical Research Institute of Murcia (IMIB-Arrixaca), E-30120 Murcia, Spain

**Keywords:** prediabetes, carob, glucose metabolism, insulin resistance, clinical trial, type 2 diabetes

## Abstract

**Background/Objectives**: A 90-day randomized double-blind and placebo-controlled study was conducted to assess the effect of carob (*Ceratonia siliqua* L.) on glucose metabolism in subjects with confirmed prediabetes. **Methods**: The carob liquid concentrate containing inositols of the carob fruit (D-pinitol, myo-inositol, D-chiro inositol) was administered at a daily dose of 6.66 g, divided into two doses of 3.33 g each. Study variables included glucose- and insulin-related parameters obtained at fasting conditions and during a standard 2 h oral glucose tolerance test (OGTT) at baseline and after 45 and 90 days of administration of the study products. **Results**: The study population included 52 subjects (25 in the experimental group, 27 in the placebo group), 27 men and 25 women, with a mean age of 45.6 ± 13.9 years. Subjects who consumed the active product showed improvements in glycated hemoglobin (HbA1c) and glucose levels as compared with placebo (*p* < 0.001 of the time × group interaction). Fasting serum insulin showed within-group significant decreases in the experimental group, with insulin indexes (HOMA-IR and QUICKI) improving significantly in the experimental group only. In the OGTT, there were significant improvements in the AUC of glucose and insulin, as well as glucose peak in the experimental group only. The product was well tolerated and no adverse effects were recorded. **Conclusions**: The use of a specific carob-based liquid concentrate decreased HbA1c and glucose levels in subjects with prediabetes, which may suggest its potential clinical relevance in the prevention of the transition from prediabetes to overt type 2 diabetes.

## 1. Introduction

Prediabetes or intermediate hyperglycemia is characterized by elevated blood glucose levels above the normal range but not high enough to meet the criteria for the diagnosis of diabetes. It is identified by increased glycated hemoglobin (HbA1c) or an abnormal oral glucose tolerance test. Subjects with prediabetes have a high risk of progressing to diabetes, with an annualized conversion rate of 5–10% [1]. The global burden of prediabetes is substantial and growing, with overall prevalence rates projected to increase from 9.1% in 2021 to 10% in 2045 [2]. A recent systematic review showed a prevalence of prediabetes ranging from 2.2% to 47.9% in Eastern Mediterranean Region countries [3]. In Spain, according to the Di@bet study, 14.8% of the adult population suffer from some form of prediabetes (impaired glucose tolerance, impaired fasting glucose or both) [4].

Type 2 diabetes mellitus (T2DM), which typically arises from prediabetes, is a chronic metabolic disorder characterized by insulin resistance and progressive β-cell dysfunction, affecting over 463 million individuals worldwide and presenting significant health and economic challenges [5]. The progression of prediabetes to overt T2DM is facilitated by well-known modifiable risk factors, particularly overweight and obesity, physical inactivity, high blood pressure, hyperlipidemia, poor dietary habits, smoking, and excessive alcohol consumption, which collectively contribute to insulin resistance and metabolic dysfunction [6,7,8,9]. Despite clinical guidelines and the introduction of new effective antidiabetic drugs, management of patients with T2DM in daily practice is often unsatisfactory due to multiple causes, such as inadequate therapeutic review and monitoring, poor adherence, clinical inertia, or healthcare system limitations [10,11,12]. Therefore, proper management of prediabetes is a crucial approach for decreasing high blood glucose levels to a normal range, preventing or delaying the transition to T2DM.

All treatment strategies in patients with prediabetes emphasize lifestyle modifications focusing on cardiovascular risk reduction, smoking cessation, healthy diet, exercise, and weight loss as cornerstones of management. Although intensive lifestyle interventions using exercise and strict caloric control have been shown to reduce the risk of developing diabetes by more than 50% in subjects with prediabetes [13,14], such intensive programs involving sustained multiple visits and in-person sessions are difficult to adopt in clinical practice [15]. Recently, the therapeutic potential of medicinal plants to effectively relieve insulin resistance and decrease blood glucose levels has been a matter of increasing interest [16,17]. Natural plant extracts containing alkaloids, glycosides, terpenoids, flavonoids, gallotannins, and other polyphenols constitute an important source of compounds with hypoglycemic activity and antioxidant effects [18,19].

Phytochemical compounds present in different extracts of the carob tree (*Ceratonia siliqua* L.) have demonstrated considerable potential as natural antidiabetic agents. It has been shown that an aqueous extract of carob leaves reduced glucose absorption in vivo and in vitro in a dose-dependent manner [20]. Also, a methanolic extract of carob pods showed inhibitory effects against amylase and glucosidase in a streptozotocin-nicotinamide-induced diabetic rat model [21]. Other experimental studies have confirmed that various carob-derived products can effectively inhibit these key enzymes involved in carbohydrate digestion. Immature carob has also been shown to reduce intestinal glucose absorption by interfering with sodium-dependent transport, thereby contributing to improvement of glycemic control [22]. Moreover, insulin regulation of carob has been associated with its high content of D-pinitol, an inositol compound with insulin-mimetic and insulin-sensitizing mechanisms [23]. All these findings support the use of carob as a promising natural source for the control of hyperglycemia, mainly due to its effects on glucose absorption and modulation of insulin activity [24]. In a recent randomized controlled nutritional trial of 20 patients with T2DM, the ingestion of a cocoa–carbon blend induced a significant modification of three exosomal miRNAs associated with an improvement of insulin sensitivity [25].

Among the bioactive inositol compounds present in carob, D-pinitol, myo-inositol, and D-chiro-inositol in particular have attracted increasing attention due to their insulin-mimetic and insulin-sensitizing properties [23,24]. However, previous studies have primarily examined the effects of different carob-related extracts rather than focusing on the contribution of inositol-rich fractions of the product, which remain insufficiently explored in subjects with prediabetes. Therefore, the present study was designed to evaluate the 90-day effects of a standardized carob-derived liquid concentrate enriched in inositols, allowing a more targeted investigation of these bioactive compounds on glucose metabolism. The hypoglycemic effect of the carob compound was evaluated in subjects with abnormal blood glucose or HbA1c levels who were not receiving antidiabetic medications in the framework of a randomized double-blind controlled trial. Also, an intervention period of 90 days was tentatively considered appropriate to detect changes in glycemic markers associated with medium-term glucose control.

## 2. Materials and Methods

### 2.1. Study Design and Participants

This was a single-center randomized, double-blind, placebo-controlled study with two parallel arms, conducted at the Health Sciences Department of Universidad Católica San Antonio de Murcia (UCAM) in the Region of Murcia, Spain. Participants were mainly recruited by advertising the study through mass media available at UCAM. The study began on 17 February 2025 and finished on 19 December 2025. Eligible subjects were Caucasian men and women aged between 18 and 65 years with a diagnosis of prediabetes according to the American Diabetes Association (ADA) [26] (fasting plasma glucose [FPG] 100 to 125 mg/dL, oral glucose tolerance test (OGTT) 140 to 199 mg/dL, HbA1c level of 5.7% to 6.4%); a body mass index (BMI) between 20 and 35 kg/m^2^; and stable nutritional habits with no body weight gain or loss of more than 5 kg in the last 10 weeks. Exclusion criteria were the use of any drug that may interfere with glucose metabolism, history of liver or renal dysfunction, alcohol consumption (>20 g/day), history of allergic hypersensitivity or poor tolerance to any component of the study product, participation in another clinical trial within the previous 3 months, pregnant or breastfeeding women, and ineligibility as judged by the investigators.

The study was approved by the Ethics Committee of Universidad Católica San Antonio (protocol code CE012515, approval date 31 January 2025) (Murcia, Spain) and was registered in ClinicalTrials.gov (NCT07379931). Written informed consent was obtained from all participants. 

### 2.2. Intervention and Study Procedures

Participants were randomly assigned (1:1) to the intervention group (dietary supplementation with a specific carob liquid concentrate (Planttech Biotechnology Spain S.L. Llíria, Valencia, Spain) or the control group (supplementation with placebo) using a computer-generated sequence (Epidat 4.1 program) by an independent researcher not involved in the study procedures. Allocation concealment was ensured by coding the study products, and both investigators and participants remained blinded to group assignment throughout the study.

The investigational product was a specific carob liquid concentrate that contains naturally occurring inositols from carob pods, standardized for its inositol content, containing 15.5% D-pinitol and 165.2 mg/g total inositols, including myo-inositol (the technical data sheet specifies bioactives composition: inositols; D-pinitol + myo-inositol > 150 g/kg; analytical method IC-HPLC). The product was manufactured under controlled conditions to ensure batch-to-batch consistency. The daily dose was 6.66 g, divided into two doses of 3.33 g each. The product was provided in sachets, to be diluted in 100 mL of water or juice and consumed 30 min before the main meals (lunch and dinner) for 90 consecutive days. All participants followed the same regimen. The placebo consisted of an aqueous formulation containing water, colorants, stabilizers, and preservatives, matched for organoleptic properties (taste, color, and texture) but without carob-derived bioactive compounds or inositols. According to its nutritional composition, it provided negligible caloric content (1 kcal/100 g) and minimal amounts of macronutrients, ensuring metabolic neutrality. The products were to be kept refrigerated (not due to degradation of the bioactive compounds, but because of the very low risk of microbiological alteration, which could cause swelling of the sachet, while remaining completely harmless). Participants were advised to maintain their routine dietary habits and physical activity and not to modify or initiate any hormonal or medical treatment during the study unless fully justified and approved by the investigators. Compliance was defined as the consumption of at least 80% of the study product (sachets taken divided by total number of sachets [*n* = 180] × 100, so that only 36 sachets corresponding to 18 days out of 90 days could be left).

The study included a recruitment visit (±10 days), a baseline visit (visit 1), a mid-study visit at day 45 (visit 2), and a final visit at 90 days (final visit). At the recruitment visit, fulfillment of the inclusion criteria was checked, written informed consent was obtained, and subjects were randomized to the study groups. At visit 1, venous blood samples were taken after 12 h fasting for laboratory analyses, which included assessment of the glycemic profile, OGTT, insulin resistance and sensitivity indexes, and lipid profile, as well as standard hematological and biochemical chemistry analyses for safety. Fasting and postprandial capillary blood samples were also collected. Anthropometric variables were also measured, and participants were given a diary card for the nutritional survey. The study product for the first half of the study period was provided. At visit 2, the same procedures as in visit 1 were performed. The returned sachets were checked, and the study product for the second half of the study was provided. Participants were asked about the occurrence of adverse effects (AEs). At visit 3, the same procedures as in visit 2 were performed except for the provision of the dietary supplement. The returned sachets at visits 2 and 3 (end of the study) were counted and checked for compliance with the study product. Participants were questioned regarding the presence of AEs, and the results of safety laboratory analyses were assessed for normality. Finally, the organoleptic characteristics of the product (odor, taste, texture, color, and aroma) were evaluated.

### 2.3. Study Variables

Glucose metabolism variables included serum HbA1c level by high-performance liquid chromatography; fasting serum glucose (clinical chemistry analyzer B400 ByoSystems) and insulin levels; fasting capillary blood glucose levels; the area under the curves (AUCs) during the 2 h, 50 g glucose, OGTT for glucose and insulin calculated using the trapezoidal rule; capillary blood glucose (Freestyle Optium Neo Set Glucose Reader, Abbott Diabetes Care Inc., Alameda, CA, USA) and insulin levels obtained at 0, 15, 30, 45, 60, 90 and 120 min; maximal glucose and insulin levels during OGTT; and increases in serum glucose and insulin levels during OGTT. Serum insulin levels were measured using a chemiluminescence-based immunoassay (BioSystems, Barcelona, Spain) on a BA 400 BioSystems analyzer, following the manufacturer’s instructions. Blood samples were stored at 2–8 °C prior to analysis in accordance with routine laboratory protocols. Insulin resistance was calculated by the Homeostatic Model Assessment of Insulin Resistance (HOMA-IR) (higher values indicate higher insulin resistance) and insulin sensitivity using the Quantitative Insulin Sensitivity Check Index (QUICKI) (higher values indicate better sensitivity, while lower values suggest significant insulin resistance).

Bias control tests included the analysis of lipid profile (cholesterol, triglycerides, low-density lipoprotein cholesterol [LDL-C], and high-density lipoprotein cholesterol [HDL-C]) (B400 ByoSystems) and anthropometric variables. Body composition was measured by bioelectrical impedance analysis (BIA) using a whole-body BIA analyzer (Tanita BC-420MA, Tanita Corp., Tokyo, Japan), which assessed weight, BMI, fat mass, and percentage of fat mass.

A diary card (24 h dietary recall) was used to collect data for a period of 3 days (two weekly days and one weekend) at the beginning and at the end of the intervention. Participants received detailed written instructions and oral guidance to ensure accurate reporting. Dietary data were analyzed with the Dietsource^®^ (v3.0) software package.

The organoleptic characteristics of the product were evaluated using a 5-point Likert scale, where 1 indicated the worst odor/taste/texture/color/aroma previously experienced and 5 indicated the best odor/taste/texture/color/aroma previously experienced.

Safety evaluation included heart rate, systolic and diastolic blood pressure (SBP, DBP), complete blood cell count, liver function tests including bilirubin, aspartate aminotransferase (AST), alanine aminotransferase (ALT), gamma-glutamyl transpeptidase (GGT) and lactate dehydrogenase (LDH) as well as serum creatinine levels and blood urea nitrogen.

### 2.4. Study Endpoints

The primary endpoint of the study was the effect of the specific carob liquid extract on serum levels of HbA1c after 90 days of consumption as compared with placebo. Secondary endpoints included changes in fasting serum glucose, capillary glucose, serum insulin, AUCs of glucose and insulin, insulin indexes, body composition, and safety parameters.

### 2.5. Statistical Analysis

The sample size was calculated a priori based on data obtained from a previous exploratory pilot study conducted by our group, in which the standard deviation (±SD) of the mean change in serum HbA1c was 0.40% in the placebo group and 0.44% in the experimental group (*n* = 10 subjects per group). According to an expected moderate between-group difference in HbA1c and using these variability estimates, a sample size of 25 subjects per group was needed for a statistical power of 80% and a two-sided significance level of 5%. To account for an anticipated dropout rate of 10%, the final sample was increased to 27 participants per group.

The analysis was based on the per-protocol (PP) dataset corresponding to those participants who completed the study at 90 days. The Shapiro–Wilk test and visual inspection of the Q-Q plots and histograms were used to assess the distribution of data, and no substantial variations from normality were observed. Moreover, sensitivity analyses using non-parametric tests, such as the Mann–Whitney *U* test and the Friedman test, yielded similar results. Categorical variables are expressed as frequencies and percentages and continuous variables as mean and ±SD. The distribution of variables between the experimental and placebo groups was compared with the chi-square test for categorical variables and Student’s *t* test for continuous variables. Changes in variables in the study groups over the course of the study were analyzed with the analysis of variance (ANOVA) for repeated measures with two study factors: within-subject factor (baseline, 45 days [mid-visit] and 90 days [end of study]) and between-subject factor (intervention: active product and placebo) for paired data. Post hoc analyses were performed with Bonferroni’s correction or Tukey’s procedure. A sensitivity analysis using the intention-to-treat (ITT) dataset (all randomized subjects) was performed for serum levels of HbA1c as the primary endpoint of the study. Missing final values for the two participants from the experimental group lost to follow-up were imputed using a conservative baseline observation carried forward (BOCF) approach. Statistical significance was set at *p* < 0.05. Reported *p* values correspond to time effects (baseline, 45 days, and 90 days) for within-group comparisons, and time × group interaction for between-group comparisons. Data were analyzed with the Statistical Package for the Social Sciences (SPSS) version 27.0 (IBM Corp., Armonk, NY, USA).

## 3. Results

### 3.1. Study Population

**Figure 1:**
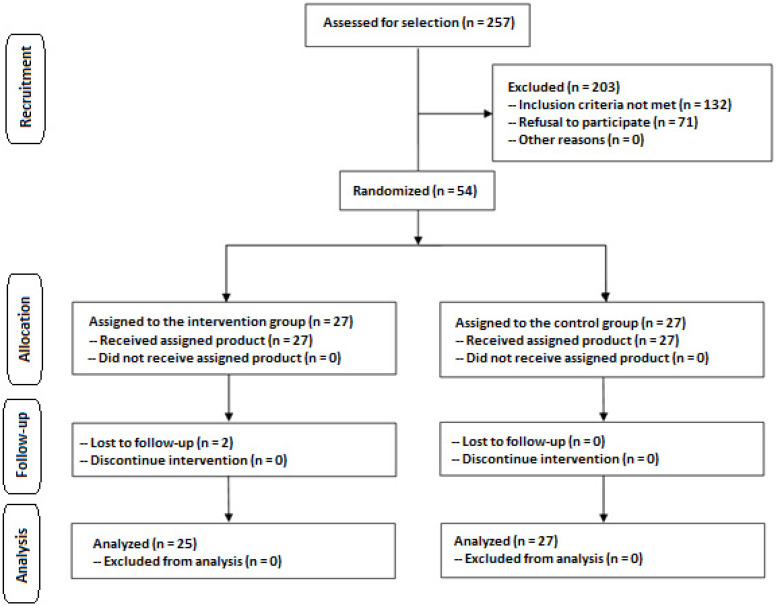
Flow chart of the study population.

Of a total of 257 subjects who were initially screened for participation, only 54 were eligible; 203 subjects were excluded because the inclusion criteria were not met (*n* = 132) or they refused to participate (*n* = 71). The remaining 54 subjects were randomized (27 to each study group), but two subjects assigned to the experimental group were lost to follow-up. Therefore, the final study population included 52 subjects (25 in the experimental group, 27 in the control group) (Figure 1).

There were 27 men and 25 women (13 men, 12 women in the experimental group; 14 men, 12 women in the placebo group). The mean age of participants was 45.6 ± 13.9 years, and the mean BMI was 26.7 ± 5.1 kg/m^2^ (Table 1).

### 3.2. Glycemic Profile

#### 3.2.1. HbAc1, Fasting Glycemia, and Insulinemia

**Figure 2:**
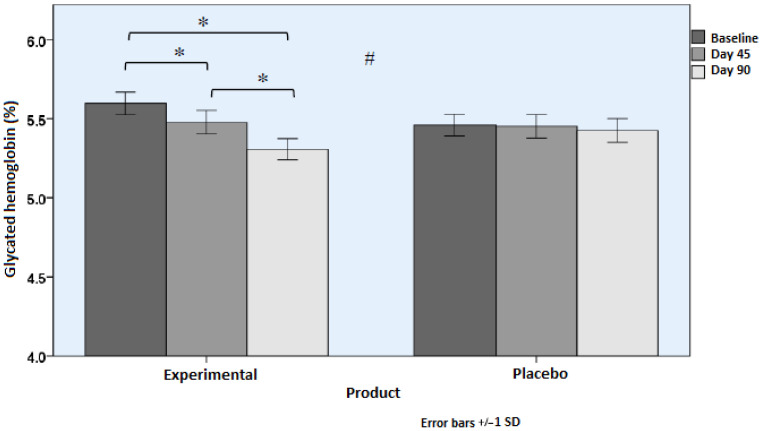
Serum levels of HbA1c (mean and SD) during the study (* *p* < 0.05 for within-group differences. # *p* < 0.05 for between-group differences [time × group interaction]).

**Figure 3:**
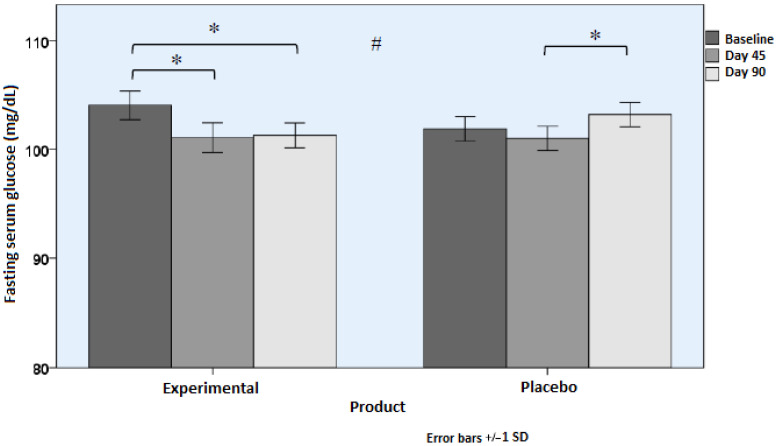
Serum levels of fasting glucose (mean and SD) during the study (* *p* < 0.05 for within-group differences; # *p* < 0.05 for between-group differences [time × group interaction]).

Changes in serum levels of HbAc1, glucose, capillary glucose, and insulin in fasting conditions are shown in Table 2. Subjects who consumed the active product showed statistically significant improvements in HbA1c (Figure 2) and glucose levels (Figure 3) as compared with subjects assigned to the placebo group (*p* < 0.001). Differences between the study groups in capillary glucose and serum insulin levels were not significant, although in the case of insulinemia, differences almost reached statistical significance (*p* = 0.071). However, in the experimental group, within-group differences were significant (*p* = 0.014).

The results of ITT sensitivity analysis for changes in serum levels of HbAc1 were consistent with results obtained in the PP population, with between-group differences of −0.23%; *p* (time × group) < 0.001.

#### 3.2.2. Results of OGTT

After a glucose load of 50 g, changes in serum levels of capillary glucose and insulin during the 2 h period of OGTT are shown in Table 3.

In relation to capillary glycemia, within-group differences at 45 and 60 min vs. baseline were statistically significant (Figure S1, Supplementary Material). Insulin levels showed within-group significant differences at 60 min vs. baseline in the placebo group and at 60, 90, and 120 min vs. baseline in the experimental group (Figure S2, Supplementary Material).

**Figure 4:**
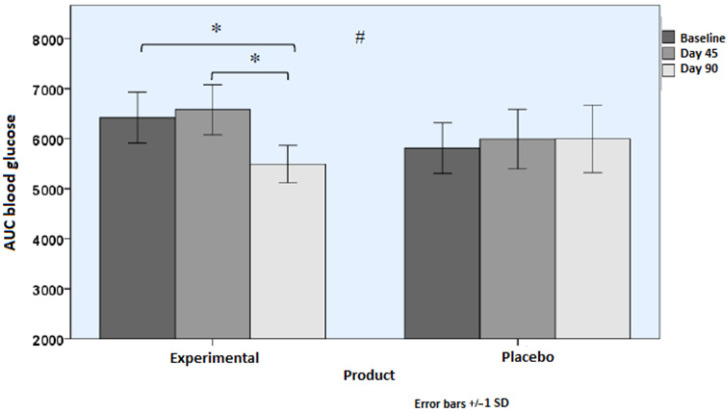
Area under the curve (AUC) of blood glucose levels during OGTT over the course of the study as compared with baseline in the two study groups (* *p* < 0.05 for within-group differences; # *p* < 0.05 for between-group differences [time × group interaction]).

AUC values for blood glucose levels at the end of the study showed a statistically significant decrease from baseline among subjects in the experimental group (*p* = 0.038), whereas changes in AUC values were not significant in the placebo group. Between-group differences were significant (*p* = 0.015) (Table 4 and Figure 4). Similar findings were observed for the AUC values of serum insulin (Table 3). Peak of blood glucose during OGTT showed statistically significant between-group differences (*p* = 0.034). Insulin peak and insulin increase levels during OGTT showed statistically significant within-group differences in the experimental group only, but between-group differences were not significant (Table 4).

### 3.3. Insulin Indexes

As shown in Table 5, both the HOMA-IR and QUICKI indexes showed statistically significant differences between the experimental and the placebo groups, with significant differences for within-group comparisons in the experimental group only. Changes in the placebo group over the course of the study were not statistically significant.

A summary of absolute changes in the study variables is shown in Table 6.

### 3.4. Lipid Profile

As shown in Table 7, statistically significant changes in serum cholesterol, triglycerides, and HDL-C during the study were not observed either in the experimental or in the control group. However, there were between-group differences in LDL-C levels (*p* = 0.003), with within-group significant increases in the placebo group.

### 3.5. Anthropometric Variables

Significant changes in anthropometric variables were not observed (Table 8).

### 3.6. Nutritional Survey

Dietary habits remained stable during the study period, and there were no differences between subjects assigned to the placebo or the experimental groups regarding nutritional variables (Table 9).

In addition, no relevant changes in physical activity were reported.

### 3.7. Organoleptic Characteristics of the Study Products

Participants in both study groups rated odor, taste, texture, color, and aroma of the study products above 3 points on a 5-point Likert scale. In the placebo group, mean values ranged between 3.11 ± 1.28 for aroma and 3.96 ±1.06 for texture, whereas in the experimental group, mean values ranged between 3.08 ± 1.04 for taste and 4.0 ± 1.08 for color.

### 3.8. Compliance and Safety

Consumption of the assigned product ranged between 80% and 100%, with a maximum number of 34 sachets returned by some participants. No abnormal changes in SBP, DBP and heart rate were observed during the study period. The safety of the investigational product was confirmed by laboratory analyses, with results of all blood and biochemical tests within normal limits.

## 4. Discussion

The administration for 90 days of a dietary supplement based on a natural, specific liquid concentrate of carob in subjects diagnosed with prediabetes was associated with an overall improvement in variables related to glucose and insulin metabolic control. These findings are clinically relevant for various reasons. Firstly, and most importantly, is the hypoglycemic effect, which may prevent or delay the progression to T2DM. Secondly, the beneficial dietary intervention is an essential component of non-pharmacological strategies in the management of prediabetes and T2DM. Thirdly, the active product showed an excellent tolerability and safety profile.

A recent pooled analysis of 19,255 subjects with prediabetes collected from prospective cohort studies conducted in Asia, Australia, Europe, North America and South Africa showed that after a median follow-up of 9.8 years, the probability of progression to T2DM was lower (12.5%) than the probability of reverting to normoglycemia (36.1%) [27]. Overweight and obesity, and decreased HDL-C concentration were risk factors that reduced prediabetes reversion to normal glycemic levels. However, in the highest fasting plasma glucose quartile, the probability of progression to T2DM increased to 16.1% and reversion decreased to 13.4% [27]. Moreover, a systematic review and meta-analysis of 59 studies to estimate risk factors for the progression from prediabetes to T2DM showed that the higher the blood glucose levels, the higher the risk of developing T2DM [28]. Therefore, efforts to decrease glucose levels, particularly in subjects with glucose levels in the higher hyperglycemic range, are necessary for achieving prediabetes transition to normoglycemia.

Consumption of the study product during the intervention period was associated with an improvement in baseline glycemic control with significant differences as compared to the placebo group in HbA1c and blood glucose levels. Also, postprandial capillary glycemia showed significant differences at 45 and 60 min, reflecting a more favorable postprandial glycemic response than in subjects assigned to the placebo arm. In relation to serum insulin levels, there were significant decreases in the experimental group only, but between-group differences did not reach statistical significance. However, in the time-blood insulin curve, significant differences at 60, 90, and 120 min were observed in the experimental group, suggesting favorable changes in the insulin metabolic dynamics. The results obtained in the AUC of glucose during the OGTT and glucose peak, with significant differences as compared with the placebo group, further confirm the hypoglycemic effect of the study product. In line with these findings, the AUC of insulin, insulin peak, and insulin increase decreased significantly in the experimental group. Improvements in insulin resistance and sensitivity, as shown by differences in the HOMA-IR and QUICKI indexes, are consistent with the beneficial effects of the carob extract on glucose metabolism.

The potential of carob components, such as D-pinitol and polyphenols, in controlling blood glucose levels and diabetes has been an interesting area of research, especially in animal models, in which consistent antidiabetic effects have been observed [28,29,30,31]. In a randomized, double-blind study, 40 healthy volunteers and 40 overweight volunteers with impaired glucose tolerance (IGT) received either a carob-pod, pinitol-enriched beverage or a sucrose-enriched beverage as a placebo for 6 weeks. An increased change in two proteins involved in the insulin secretion pathway was observed in the serum proteomic profile of the IGT group, leading the authors to suggest that the naturally based carob-pod, pinitol-enriched beverage could help reduce blood glucose levels by protecting β-cells and stimulating the insulin secretion pathway [32].

In human investigations, studies in healthy subjects have reported improvements in postprandial glycemia and insulin sensitivity after short-term consumption of carob extracts [33,34,35]. In a recent study of the administration of two commercial carob syrups to healthy volunteers, both products displayed statistical significance in low glycemic indices and attenuation of the postprandial glucose response by 16% compared to glucose control, as well as statistically significant reduction in total cholesterol and waist circumference after a 6-week consumption period [36]. In a 12-week double-blind clinical trial performed in 40 healthy subjects administered either an inositol-enriched carob extract or a sucrose-sweetened beverage twice a day, analysis of postprandial glucose levels at breakfast, lunch and dinner revealed a mean reduction in glucose of ≈14% and a significant reduction in the area under the curve at 24 h after consumption of inositol-enriched carob product [35]. However, the effect of consumption of a carob extract in subjects with prediabetes or T2DM in the framework of a randomized controlled trial has not been previously examined.

In relation to changes in lipid profile and anthropometric variables as bias control parameters, serum triglycerides decreased significantly in the experimental group, whereas LDL-C levels increased significantly in the placebo group. Anthropometric parameters measured by BIA did not vary significantly throughout the study. Relevant changes in the dietary patterns of participants were not recorded. The study product was also well-tolerated and did not cause adverse effects. Likewise, compliance with treatment was adequate, and the organoleptic characteristics of the study products were also satisfactorily rated.

The present findings, however, should be interpreted taking into account the limitations of the study, including the single-center design, reduced sample size, and the short intervention period of 90 days. However, the design of the investigation as a randomized double-blind placebo-controlled study supports the validity of the efficacy of the carob extract for improving HbA1c and glucose levels in subjects with a definitive diagnosis of prediabetes. Finally, some methodological aspects should be considered. In the metabolic/experimental context of the study, the 50 g oral glucose load test is consistent with standardized protocols for the assessment of postprandial glycemic responses, as established by the FAO/WHO Joint Expert Consultation on Carbohydrates in Human Nutrition [37], rather than the standard 75 g oral glucose load of the OGTT for diagnostic purposes. The primary analysis was conducted in the PP dataset, as only two participants from the experimental group were lost to follow-up. Given this very low attrition rate (≈3.8% of the randomized sample), the risk of bias associated with missing data is minimal and unlikely to have affected the results. Although the investigational product was standardized in its inositol content, a more detailed characterization of its composition would further improve the reproducibility of the study findings. Regarding the potential effect of physical activity, although participants reported no relevant changes during the study period, specific tools for assessing physical activity, such as validated questionnaires, were not used. However, given the stability of dietary intake observed during the study, it may be assumed that metabolic outcomes are unlikely to be influenced by modifications in lifestyle factors.

## 5. Conclusions

In this randomized controlled trial, the administration of a specific carob-derived liquid concentrate for 90 days in subjects with prediabetes was associated with improvements in several markers of glucose metabolism, including HbA1c, fasting glucose, and postprandial responses, although these findings should be interpreted with caution given the small study population and the short duration of the intervention. These results suggest a possible beneficial effect of the carob liquid extract on glycemic control, but further large-scale and long-term randomized studies are needed to confirm the present findings and to determine their clinical relevance.

## Figures and Tables

**Table 1:** Age and anthropometric data of the study population.

Data	Placebo (*n* = 27)	Experimental (*n* =25)	Total (*n* = 52)
Age, years	45.0 ± 16.5	46.5 ± 11.1	45.6 ± 13.9
Height, cm	169.9 ± 9.6	170.6 ± 10.2	170.7 ± 9.8
Weight, kg	78.5 ± 18.0	78.8 ± 18.5	78.7 ± 17.5
Body mass index (BMI), kg/m^2^	26.5 ± 5.5	27.0 ± 5.1	26.7 ± 5.1

**Table 2:** Serum levels of HbAc1, glucose, and insulin during the study period.

Variables	Baseline	Mid-Study (45 Days)	Final (90 Days)	Within-Group *p* Value	*p* Value (Time × Group)
HbA1c, %					
Placebo	5.5 ± 0.4	5.5 ± 0.4	5.4 ± 0.4	0.585	<0.001
Experimental	5.6 ± 0.4	5.5 ± 0.4	5.3 ± 0.3	<0.001	
Fasting serum glucose, mg/dL					
Placebo	101.9 ± 5.9	101.0 ± 5.8	103.2 ± 5.8	0.367	<0.001
Experimental	104.1 ± 6.6	101.1 ± 6.8	101.3 ± 5.7	<0.006	
Fasting capillary glucose, mg/dL					
Placebo	98.3 ± 9.3	97.2 ± 9.5	96.6 ± 7.1	0.982	0.713
Experimental	95.8 ± 7.5	96.4 ± 6.6	95.4 ± 5.3	1.0	
Fasting serum insulin, IU/mL					
Placebo	9.5 ± 2.2	9.2 ± 3.6	9.5 ± 2.5	1.0	0.071
Experimental	10.4 ± 2.6	10.3 ± 3.5	8.7 ± 3.5	0.014	

**Table 3:** Capillary glucose and insulin levels at different time points during OGTT.

Parameter	Time Points	Placebo Group		Experimental Group	
		Baseline	Final	Baseline	Final
Capillary glucose level (mg/dL)	Glucose pre-load	98.3 ± 9.3	96.6 ± 7.1	95.9 ± 7.5	95.4 ± 5.3
	15 min	142.6 ± 21.8	143.5 ± 22.4	150.2 ± 14.8	145.3 ± 18.8
	30 min	177.4 ± 27.8	170.9 ± 30.5	173.7 ± 20.2	168.6 ± 21.7
	45 min	178.3 ± 33.0	176.4 ± 42.8	182.7 ± 27.4	169.2 ± 24.8
	60 min	169.1 ± 40.0	168.0 ± 50.0	177.6 ± 35.3	161.9 ± 29.0
	90 min	131.7 ± 40.1	136.7 ± 42.6	132.3 ± 35.4	129.5 ± 25.3
	120 min	99.9 ± 34.3	103.0 ± 34.0	102.4 ± 26.1	94.3 ± 17.5
Insulin level (IU/mL)	Glucose pre-load	11.2 ± 3.6	11.2 ± 2.9	9.8 ± 1.9	10.2 ± 2.9
	15 min	38.5 ± 14.0	41.2 ± 13.0	33.3 ± 15.9	29.5 ± 7.9
	30 min	51.0 ± 21.1	55.6 ± 17.3	49.0 ± 25.0	43.66 ± 12.2
	45 min	69.5 ± 33.6	67.5 ± 25.1	60.7 ± 20.8	55.3 ± 12.9
	60 min	71.1 ± 38.2	67.9 ± 25.0	69.5 ± 18.3	53.5 ± 12.2
	90 min	40.0 ± 21.6	40.8 ± 18.0	53.9 ± 20.8	44.9 ± 18.1
	120 min	16.3 ± 12.9	19.8 ± 11.8	34.9 ± 17.2	27.2 ± 12.9

OGTT: oral glucose tolerance test.

**Table 4:** Glucose and insulin parameters recorded at OGTTs during the study visits.

Variables	Baseline	Mid-Study (45 Days)	Final (90 Days)	Within-Group *p* Value	*p* Value (Time × Group)
AUC glucose, mmol/L·min					
Placebo	5813.2 ± 2636.3	5990.8 ± 3078.2	5995.5 ± 3516.5	1.0	0.015
Experimental	6419.3 ± 2557.5	6581.2 ± 2493.1	5494.3 ± 1888.1	0.038	
Glucose peak, mg/dL					
Placebo	186.3 ± 31.4	182.7 ± 37.2	184.6 ± 44.3	1.0	0.034
Experimental	189.1 ± 27.5	191.9 ± 26.0	180.2 ± 22.9	0.121	
Glycemia increase, mg/dL					
Placebo	88.1 ± 28.0	87.1 ± 34.7	88.0 ± 42.8	1.0	0.137
Experimental	93.4 ± 28.1	95.2 ± 25.2	84.9 ± 21.9	0.289	
AUC insulin, IU/mL·min					
Placebo	4157.5 ± 2420.6	4268.7 ± 1198.8	4255.0 ± 1579.2	1.0	0.019
Experimental	4748.9 ± 1602.0	3868.6 ± 1196.8	3739.9 ± 1145.7	0.031	
Insulin peak, IU/mL					
Placebo	77.0 ± 35.3	71.9 ± 15.5	73.8 ± 23.4	1.0	0.165
Experimental	74.3 ± 20.4	62.9 ± 15.2	59.0 ± 13.1	0.031	
Insulin increase, IU/mL					
Placebo	65.8 ± 36.1	60.8 ± 16.0	62.6 ± 23.7	1.0	0.155
Experimental	64.5 ± 19.6	52.7 ± 14.2	48.8 ± 12.5	0.030	

OGTT: oral glucose tolerance test; AUC: area under the curve.

**Table 5:** Changes in insulin indexes during the study.

Variables	Baseline	Mid-Study (45 Days)	Final (90 Days)	Within-Group *p* Value	*p* Value (Time × Group)
HOMA-IR					
Placebo	2.39 ± 0.57	2.30 ± 0.94	2.41 ± 0.63	1.0	0.038
Experimental	2.69 ± 0.73	2.59 ± 1.00	2.20 ± 0.93	0.006	
QUICKI					
Placebo	0.34 ± 0.01	0.34 ± 0.03	0.34 ± 0.01	1.0	0.024
Experimental	0.33 ± 0.02	0.34 ± 0.02	0.35 ± 0.03	0.002	

HOMA-IR: Homeostatic Model Assessment of Insulin Resistance; QUICKI: Quantitative Insulin Sensitivity Check Index.

**Table 6:** Absolute changes in glycemic-related variables in the two study groups.

Study Variables	Experimental Group		Placebo Group	
	Absolute Change	95% Confidence Interval	Absolute Change	95% Confidence Interval
HbA1c	−0.29	−0.36; −0.22	−0.04	−0.10; 0.03
Fasting capillary glucose	−0.4	−4.7; 3.9	−1.7	−5.8; 2.5
Fasting serum glucose	−2.8	−4.9; −0.7	1.3	−0.7; 3.3
AUC glucose	−925	−1810; −39	182	−669; 1034
Glucose peak	−8.9	−19.3; 1.6	−1.7	−11.8; 8.3
Fasting serum insulin	−1.7	−3.2; −0.3	−0.02	−1.41; 1.37
AUC insulin	−1009	−1947; −71	98	−805; 1000
Insulin peak	−15.3	−29.6; −1.1	−3.2	−16.9; 10.5
HOMA-IR	−0.5	−0.9; −0.1	0.2	−0.3; 0.4
QUICKI	0.01	0.00; 0.02	0.00	−0.01; 0.01

**Table 7:** Changes in the lipid profile in the placebo and experimental groups during the study.

Variables	Baseline	Mid-Study (45 Days)	Final (90 Days)	Within-Group *p* Value	*p* Value (Time × Group)
Serum cholesterol, mg/dL					
Placebo	182.2 ± 41.5	182.7 ± 42.3	190.9 ± 42.0	1.0	0.149
Experimental	201.9 ± 36.4	204.0 ± 28.5	202.7 ± 27.8	1.0	
Serum triglycerides, mg/dL					
Placebo	110.0 ± 66.8	105.2 ± 64.1	116.3 ± 79.0	1.0	0.024
Experimental	119.6 ± 57.3	116.7 ± 62.6	115.9 ± 58.0	0.002	
LDL-C, mg/dL					
Placebo	110.4 ± 33.0	110.6 ± 36.9	120.3 ± 32.7	0.025	0.003
Experimental	123.9 ± 36.3	126.4 ± 26.1	121.1 ± 27.1	1.0	
HDL-C, mg/dL					
Placebo	49.8 ± 14.8	51.0 ± 12.3	49.9 ± 12.7	1.0	0.852
Experimental	53.2 ± 13.5	53.7 ± 13.5	53.0 ± 13.5	1.0	

LDL-C: low-density lipoprotein cholesterol; HDL-C: high-density lipoprotein cholesterol.

**Table 8:** Changes in anthropometric variables in the placebo and experimental groups during the study.

Variables	Baseline	Mid-Study (45 Days)	Final (90 Days)	Within-Group *p* Value	*p* Value (Time × Group)
Body weight, kg					
Placebo	81.8 ± 12.4	81.7 ± 12.6	81.4 ± 12.4	1.0	0.082
Experimental	79.5 ± 13.0	80.0 ± 13.1	79.8 ± 13.0	1.0	
Body mass index (BMI), kg/m^2^					
Placebo	28.2 ± 3.8	28.1 ± 3.9	28.0 ± 3.9	1.0	0.090
Experimental	27.3 ± 3.5	27.4 ± 3.6	27.3 ± 3.5	1.0	
Fat mass, %					
Placebo	28.9 ± 7.2	29.0 ± 7.6	29.0 ± 8.0	1.0	0.077
Experimental	31.6 ± 8.0	30.8 ± 8.0	30.9 ± 8.0	0.196	
Fat mass, kg					
Placebo	23.8 ± 7.4	24.3 ± 8.0	23.9 ± 8.1	1.0	0.266
Experimental	25.1 ± 7.5	24.8 ± 7.6	24.7 ± 7.5	0.876	

**Table 9:** Changes in energy intake, carbohydrates, lipids, and proteins during the study.

Variables	Placebo Group		Experimental Group		*p* Value (Time × Group)
	Baseline	Final	Baseline	Final	
Energy intake, kcal/day	2148 ± 382	2121 ± 401	2189 ± 367	2162 ± 389	0.814
Carbohydrates, g/day	236.4 ± 48.7	231.8 ± 46.9	241.2 ± 44.5	235.6 ± 47.1	0.779
Lipids, g/day	82.1 ± 18.4	80.7 ± 17.9	84.6 ± 16.8	83.2 ± 17.5	0.801
Proteins, g/day	92.8 ± 19.6	91.5 ± 18.7	95.1 ± 17.9	93.8 ± 18.3	0.836

## Data Availability

Data are available from the corresponding author upon request.

## Supplementary Materials

The following supporting information can be downloaded at: https://www.mdpi.com/article/10.3390/nu18101521/s1, Figure S1: Mean (±SD) capillary glucose values (mg/dL) at different time points during the 2 h OGTT in the placebo and experimental groups (* *p* < 0.05 for within-group comparisons at 45 and 60 min vs. baseline). Figure S2: Mean (±SD) venous insulin values (IU/mL) at different time points during the 2 h OGTT in the placebo and experimental groups (* *p* < 0.05 for within-group comparisons at 60 min vs. baseline in the placebo group and at 60, 90, and 120 min vs. baseline in the experimental group).

## Abbreviations

The following abbreviations are used in this manuscript:

ADA	American Diabetes Association
AE	Adverse effect
ALT	Alanine aminotransferase
AST	Aspartate aminotransferase
AUC	Area under the curve
BIA	Bioelectrical impedance analysis
BMI	Body mass index
FPG	Fasting plasma glucose
GGT	Gamma-glutamyl transpeptidase
Hb1Ac	Glycated hemoglobin
HDL-C	High-density lipoprotein cholesterol
HOMA-IR	Homeostatic Model Assessment of Insulin Resistance
IGT	Impaired glucose tolerance
LDH	Lactate dehydrogenase
LDL-C	Low-density lipoprotein cholesterol
OGTT	Oral glucose tolerance test
QUICKI	Quantitative Insulin Sensitivity Check Index
SD	Standard deviation
T2DM	Type 2 diabetes mellitus
