# Supplementary figures and images for: Effects of Specific Carob (Ceratonia siliqua L.) Liquid Concentrate on Glucose Metabolism in Subjects with Prediabetes: A Randomized Double-Blind Controlled Clinical Trial
Source: Nutrients. 2026 May 10;18(10):1521. doi: 10.3390/nu18101521 (PMC13210344; doi:10.3390/nu18101521)

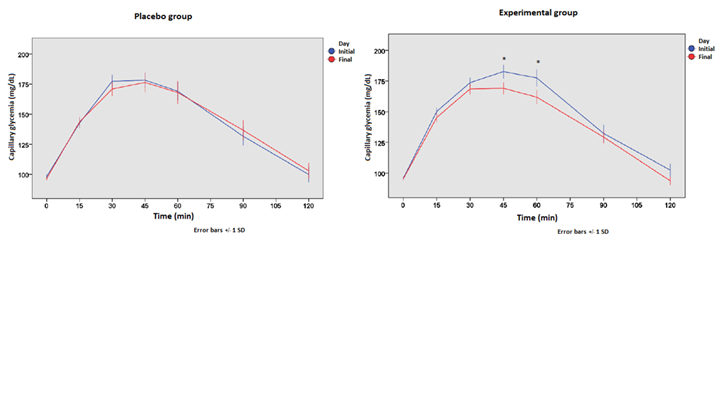

Supplement: Supplementary file 1 [file nutrients-18-01521-s001.zip › S1.CapillaryGlu.tif]

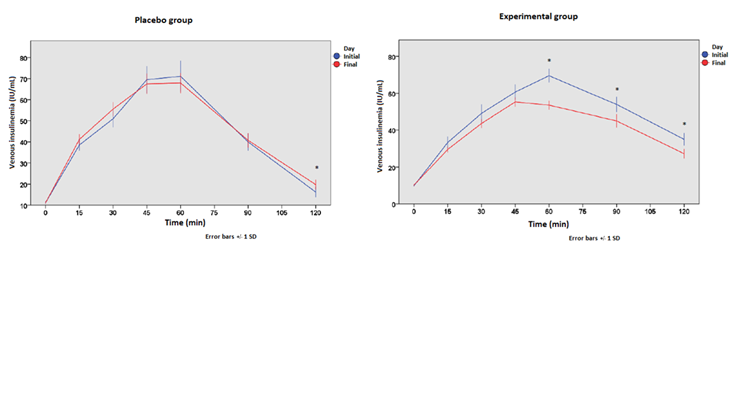

Supplement: Supplementary file 1 [file nutrients-18-01521-s001.zip › S2.Venus_Insu.tif]
